# Effective-mass model and magneto-optical properties in hybrid perovskites

**DOI:** 10.1038/srep28576

**Published:** 2016-06-24

**Authors:** Z. G. Yu

**Affiliations:** 1 ISP/Applied Sciences Laboratory, Washington State University, Spokane, Washington 99210, USA

## Abstract

Hybrid inorganic-organic perovskites have proven to be a revolutionary material for low-cost photovoltaic applications. They also exhibit many other interesting properties, including giant Rashba splitting, large-radius Wannier excitons, and novel magneto-optical effects. Understanding these properties as well as the detailed mechanism of photovoltaics requires a reliable and accessible electronic structure, on which models of transport, excitonic, and magneto-optical properties can be efficiently developed. Here we construct an effective-mass model for the hybrid perovskites based on the group theory, experiment, and first-principles calculations. Using this model, we relate the Rashba splitting with the inversion-asymmetry parameter in the tetragonal perovskites, evaluate anisotropic *g*-factors for both conduction and valence bands, and elucidate the magnetic-field effect on photoluminescence and its dependence on the intensity of photoexcitation. The diamagnetic effect of exciton is calculated for an arbitrarily strong magnetic field. The pronounced excitonic peak emerged at intermediate magnetic fields in cyclotron resonance is assigned to the 3*D*_±2_ states, whose splitting can be used to estimate the difference in the effective masses of electron and hole.

Hybrid organic-inorganic perovskites such as CH_3_NH_3_PbI_3_ represents a revolutionary breakthrough for low-cost solar cells^1^,^2^,^3^,^4^ because of their desirable optical and carrier transport properties^5^,^6^. The materials also exhibit many intriguing features, including strong spin-orbit coupling^7^ and the associated Rashba effect^8^,^9^, large-radius Wannier excitons^10^, and novel magnetic-field effect (MFE) in photoluminescence (PL) and photoconduction^11^,^12^, and show promise in light-emitting^13^ and thermoelectric^14^ applications.

These outstanding properties are interconnected and are ultimately determined by the material’s unusual electronic structure, which has been intensively studied by a variety of density-functional calculations^15^,^16^,^17^. While such first-principles calculations are indispensable in predicting the crystal structure, carrier effective mass, and band gap, they become increasingly unwieldy in studying processes involving excited states and under external fields. An alternative is to develop an effective-mass Hamiltonian^18^,^19^,^20^, which is both tractable and transparent in physics with parameters determined by experimentally measured properties. These properties, such as effective masses and *g*-factors, are usually obtained from magneto-optical studies, which turn out to be a major experimental means of validating the principle of the band theory of semiconductors.

Exciton, an electron-hole pair bounded by the Coulomb interaction, is a fundamental excitation in semiconductors. The exciton binding energy in the hybrid perovskites is critical to photovoltaic and light-emitting efficiencies and has been a subject of intense debate^21^,^22^. This controversy can be resolved via a definitive measurement of magneto-optical absorption (cyclotron resonance)^23^, which reveals characters of exciton as well as constituent electron and hole. To take advantage of the wealth of information, a detailed analysis of cyclotron resonance is needed. While diamagnetic response of an exciton is similar to that of a hydrogen, a key difference is that the electron and hole in an exciton have a comparable effective mass, particularly in the hybrid perovskites.

A moderate magnetic field less than 1 T is found to be able to influence exciton PL in CH_3_NH_3_PbI_3_^11^,^12^, indicating that magnetic field is a versatile tool for studying excitons and free carriers. This MFE has been attributed to the Δ*g* (the difference between electron and hole *g*-factors) mechanism, frequently encountered in organic radical pairs^24^. However, the lack of knowledge on the *g*-factors in CH_3_NH_3_PbI_3_ hampers the development of a clear understanding of the MFE. In addition, the MFE is sensitive to the intensity of photoexcitation^12^, which is not well understood.

Here we construct an effective-mass model of CH_3_NH_3_PbI_3_ based on information available in literature. This model, which can be extended to other hybrid perovskites by using suitable parameters, reveals connections among the *g*-factors, effective masses, and Rashba spin splittings. Using this model, we examine the MFE on exciton PL and find that the MFE is controlled by the interplay of exchange energy, exciton (spin) relaxation time, and the Zeeman energy. Besides the Δ*g* mechanism, a Σ*g* (summation of the electron and hole *g*-factors) mechanism can manifest itself in the MFE. The dependence of MFE on the intensity of photoexcitations is quantitatively explained in terms of the screening effect by the photogenerated carriers, which greatly reduces the exchange coupling of excitons. The diamagnetic effect on excitons under an arbitrarily large magnetic field is reliably calculated and in an excellent agreement with recent cyclotron measurements. The experimentally observed pronounced excitonic absorption peak, induced by the magnetic-field, can be attributed to the 3*D*_±2_ states, whose energy splitting can be used to determine the difference in electron and hole effective masses. Our results demonstrate the efficacy of the effective-mass model in understanding magneto-optical properties and suggest it a foundation for systematically studying many other transport, optical, and spintronic processes in the hybrid perovskites.

## Results

### Model

Crystalline CH_3_NH_3_PbI_3_ can have the high-temperature *α*-phase with the pseudo cubic (*O*_*h*_) symmetry, the intermediate-temperature *β*-phase with the tetragonal (*C*_4*v*_) symmetry, and the low-temperature orthorhombic *γ* phase. Phase transitions from high to low temperatures, being of group-subgroup type, take place at 333 K and 150 K, respectively^25^,^26^. We focus in this paper on the *β*-phase, which is also a good description of the approximately uniaxial *γ*-phase^25^. In the *β*-phase, PbI_6_ octahedra are misalign with the C-axis (symmetry axis of *C*_4*v*_), and the structure is noncentrosymmetric.

First-principle calculations indicate that the valence and conduction bands are mainly associated with cationic (Pb) *s* and *p* orbitals, respectively^15^,^16^,^17^,^27^, denoted as *S*, *X*, *Y*, and *Z*. The direct band gap is located at *R* point^7^, which has the same *C*_4*v*_ symmetry as the crystal structure. Since physically relevant states are those close to the band extremes, we derive band structure in the neighborhood of *R* point, via the ***k*** **·** ***p*** method, where ***k*** is the wave vector away from the *R* point. In this method, the wave function at ***k*** is expressed as *ψ*_*n**k***_ = *e*^*i**k*****·*****r***^*u*_*n*_(***r***) with *u*_*n*_(***r***) being the basis function of *n*th band at the *R* point. We note that the ***k*** **·** ***p*** Hamiltonian for zinc-blende semiconductors is not suitable for the tetragonal perovskites^15^. In the absence of magnetic field, the Hamiltonian can be written as *H* = *H*_0_ + *H*_*SO*_, where *H*_0_ = *p*^2^/2 *m* + *V*(***r***) is spin-independent part and 
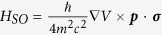
 is the spin-orbit coupling (SOC). Here *V*(***r***) is periodic potential, ***p*** is momentum, *m* is free electron mass, *c* is the speed of light, and ***σ*** are the Pauli matrices.

In the *β*-phase, the potential should be an identical representation of group *C*_4*v*_, a subgroup of the cubic group *O*_*h*_, and therefore can be expressed in terms of the *O*_*h*_ representations, in particular, its first three irreducible representations, Γ_1_ ⊕ Γ_12_ ⊕ Γ_15_. Neglecting the trivial Γ_1_ representation, we write *V*(***r***) = ∑_*j*_*c*_*j*_*d*_*j*_, where *d*_1_ = 2*z*^2^ − *x*^2^ − *y*^2^ and *d*_2_ = *x*^2^ − *y*^2^ are the basis functions of Γ_12_, and *d*_3_ = *x*, *d*_4_ = *y*, and *d*_5_ = *z* are the basis functions of Γ_15_. By requiring *D*(*G*_*i*_)*V*(***r***) = *V*(***r***) with *G*_*i*_ being the symmetry operators in *C*_4*v*_, *c*_2_ = *c*_3_ = *c*_4_ = 0. The nonzero *c*_1_ reflects a crystal-field splitting between *Z* and *X* (*Y*), 〈*X*|*H*_0_|*X*〉 = 〈*Y*|*H*_0_|*Y*〉 = −〈*Z*|*H*_0_|*Z*〉/2 = *δ*/3. And *c*_5_ originates from the lack of inversion asymmetry in *C*_4*v*_, giving rise to^28^ 〈*S*|*H*_0_|*Z*〉 = 〈*Z*|*H*_0_|*S*〉^*^ = *c*_5_〈*S*|*z*|*Z*〉 ≡ *ζ*.

The Hamiltonian *H*_0_, up to the second order of *k*, can be written as^19^,^20^


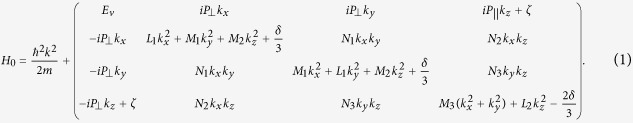


Here *E*_*v*_ is the valence-band maximum, *L*_*i*_, *M*_*i*_, and *N*_*i*_ are parameters due to the interaction between the conduction bands with far bands other than the valence band, and 
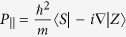
 and 
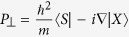

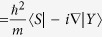
 are the Kane parameters that connect the valence-band and conduction-band orbitals^20^.

The SOC among the *p* orbitals mixes up and down spins, *λ* = *i*〈*X*↑|*H*_*SO*_|*Y*↓〉 = *i*〈*Y*↑|*H*_*SO*_|*Z*↓〉 = *i*〈*Z*↑|*H*_*SO*_|*X*↓〉, which is particularly strong in the hybrid perovskites due to the heavy element Pb, *λ* = 1.2–1.5 eV^7^,^8^,^9^, and cannot be treated as a perturbation.

**Figure 1:**
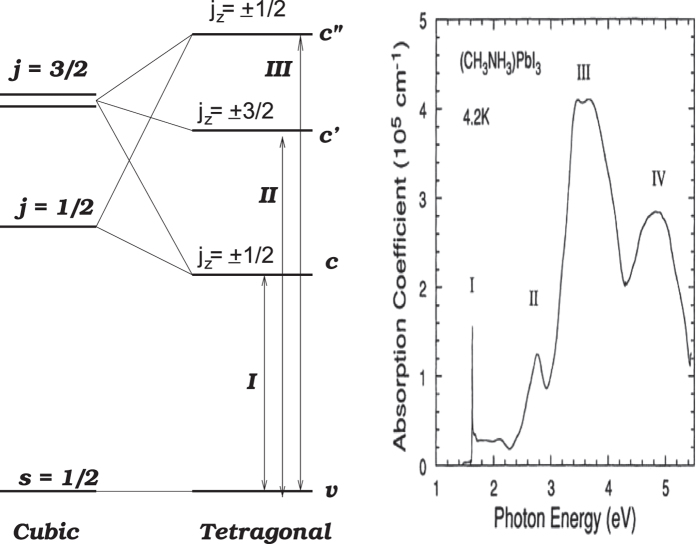
Schematic diagram of band edges and their angular momenta in CH_3_NH_3_PbI_3_. The optical absorption spectrum is adapted from ref. 29.

The crux of the ***k*** **·** ***p*** method is that the basis functions of *u*_*n*_(***r***) should be the eigenstates of *H* at *k* = 0^20^, which can be achieved by choosing the following basis functions *u*_*n*_(***r***),


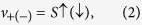



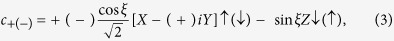



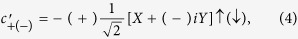



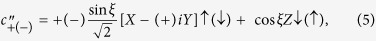


with 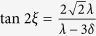. The angular momentum is *s* = 1/2 for the valence band *v*_±_, and *j* = 1/2 (***j*** = ***l*** + ***s*** with *l* = 1 and *s* = 1/2) for the first conduction band *c*_±_. The two upper conduction bands, 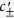, and 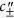, have *j* = 3/2, with *j*_*z*_ = ±3/2 for 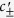 and *j*_*z*_ = ±1/2 for 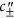. The diagonal elements at *k* = 0 in these basis functions are *E*_*v*_, 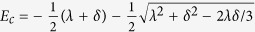, *E*_*c*′_ = 0, 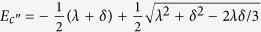. Here we temporally neglect *ζ*, which is small as compared to other parameters. In the measured absorption spectra of CH_3_NH_3_PbI_3_ (Fig. 1), the first three peaks, located at 1.6 eV, 2.8 eV, and 3.4 eV^29^, can be attributed to electron transitions from the valence band to the three conduction bands. Thus we obtain the values *E*_*v*_ = −2.8 eV, *E*_*c*_ = −1.2 eV, and *E*_*c*′_ = 0.6 eV, which fix the parameter values, *λ* = 1.4 eV, *δ* = −0.7 eV, and sin *ξ* = 0.411.

An applied magnetic field ***B*** have two effects on a charge carrier: paramagnetic magnetism due to the carrier’s spin and diamagnetic orbital magnetism due to the lack of commutation among momentum components, 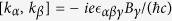
30, where 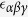 is the antisymmetric tensor of rank three. Because of the latter, the effective Zeeman energy of quasi-degenerate conduction-band orbitals can be written as


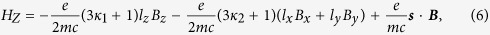


where ***l*** (*l* = 1) is the angular momentum operator, and *κ*_1_ and *κ*_2_ are the Luttinger antisymmetric parameters for tetragonal structures.

The total Hamiltonian, in the presence of magnetic field ***B***, can now be written as


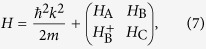


where 4 × 4 matrices *H*_A_, *H*_B_ and *H*_C_ are displayed in the Methods section. In this Hamiltonian, we have eleven parameters, *L*_*i*_ and *κ*_*i*_ (*i* = 1, 2), *M*_*i*_ and *N*_*i*_ (*i* = 1, 2, 3), 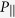, and *P*_⊥_. If the information at hand is insufficient to fix all these parameters, one can resort to the first principles calculations, or via the quasi-cubic symmetry, *L*_1_ = *L*_2_, *M*_1_ = *M*_2_ = *M*_3_, and *κ*_1_ = *κ*_2_.

### *g*-factors, effective masses, and cyclotron frequencies

The most important transport and optical processes occur in the conduction and valence bands, which are both nondegenerate (excluding spin). We map the above 8 × 8 Hamiltonian *H*_*mn*_ into two effective 2 × 2 Hamiltonians in spin space for these two bands by employing the Löwdin method for degenerate perturbation theory^31^,


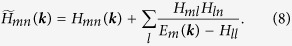


Here *E*_*m*_(***k***) is the energy of *m*th band, which, for small ***k***, can be approximated by the band-edge value. Taking into account the non-commutative relations among ***k*** components, we obtain the effective Hamiltonians, up to the second order of *k*, for the valence and conduction bands, with basis functions of *v*_±_ and *c*_±_ in Eqs (2) and (3),


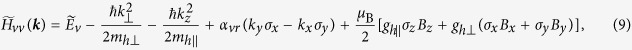



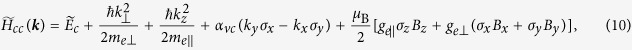


where *μ*_B_ ≡ *eħ*/(2 *mc*) is the Bohr magneton, 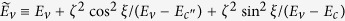 and 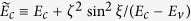, which can be approximated by *E*_*v*_ and *E*_*c*_.

The effective masses of valence band along and perpendicular to the C-axis in Eq. (9) are expressed as


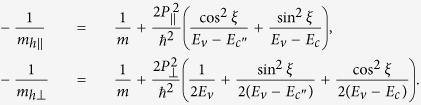


It is interesting to note that *m*_*h*||_ (*m*_*h*⊥_) depends on the interaction with the conduction bands via the Kane parameter *P*_||_ (*P*_⊥_). Similarly, the effective masses of the conduction band along and perpendicular to the C-axis are


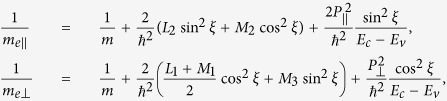


which are influenced by the interaction with the valence band, as well as by the symmetric parameters *L*_*i*_ and *M*_*i*_, stemming from the interaction with far bands. The anisotropic effective masses for the valence and conduction bands have been extensively calculated by several first-principles approaches^16^,^17^. One of the most accurate values are given in ref. 17, which are also consistent with recent cyclotron resonance measurements^23^, 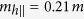, *m*_*h*⊥_ = 0.23 *m*, *m*_*e*||_ = 0.15 *m*, and *m*_*e*⊥_ = 0.21 *m*. From these effective masses, we obtain the Kane parameters, *P*_||_ = 7.64 eV Å and *P*_⊥_ = 6.95 eV Å, as well as the symmetric parameters *mL*_*i*_/*ħ*^2^ = −25.90 and *mM*_*i*_/*ħ*^2^ = 23.39.

A free electron possesses a magnetic moment of its spin and has a *g*-factor of *g*_0_ = 2.0023. The SOC enables the electron orbital motion to contribute to the magnetic moment and, consequently, the effective *g*-factor deviates from *g*_0_. In Eq. (9), the *g*-factors of the valence-band edge along and perpendicular to the C-axis are


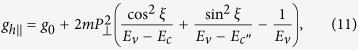



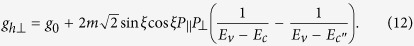


We see that the *g*-factors depend on the energies of conduction-band edges as well as the Kane parameters. In contrast to the effective mass, *g*_*h*||_ is connected to the conduction bands only via *P*_⊥_. This is understandable because a magnetic field ***B*** affects the electron orbital motion perpendicular to the field. For the same reason, *g*_*h*⊥_ is connected to the conduction bands via both *P*_||_ and *P*_⊥_. Using the values of *P*_||_ and *P*_⊥_, we obtain the *g*_*h*||_ = −0.472 and *g*_*h*⊥_ = −0.354, which are similar to the value in 2H-PbI_2_, *g*_*h*_ = −0.4^32^. The negative *g*-factor means that the up spin has a lower energy than the down spin.

The conduction-band *g*-factors along and perpendicular to the C-axis are


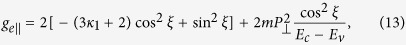



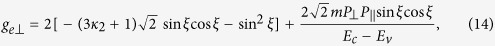


which depend on the antisymmetric Luttinger parameters *κ*_1_ and *κ*_2_, in addition to the 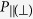. Thus the values of *g*_*e*||_ and *g*_*e*⊥_ can be used to determine *κ*_1_ and *κ*_2_. Experimentally, the exciton *g*-factor are measured from the energy splitting between left- and right-circularly polarized absorption^33^,^34^ and PL^11^, which, as we will discuss below, is *g*_*e*||_ + *g*_*h*||_. If we use *g*_*e*||_ + *g*_*h*||_ = 1.2, as in ref. 33, we find *g*_*e*||_ = 1.672 and *κ*_1_ = 0.269. If we further assume *κ*_2_ = *κ*_1_, we have *g*_*e*⊥_ = 2.281. These values are also similar to those of electrons in 2H-PbI_2_, *g*_*e*||_ = 1.4 and *g*_*e*⊥_ = 2.4^32^.

Since both the *g*-factors and the effective masses are anisotropic, the effective spin splitting and the cyclotron frequency depend on the angle *θ* between the magnetic field and the C-axis,


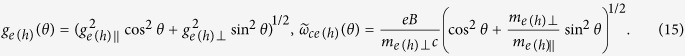


**Figure 2:**
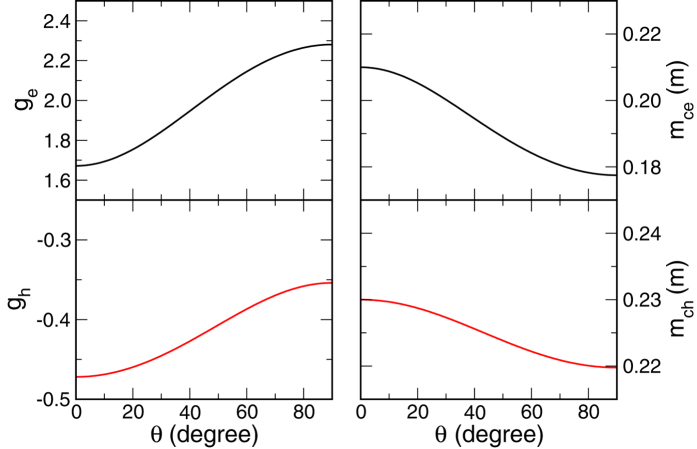
*g*-factors and cyclotron masses of conduction and valence bands. Left panels describe the electron (*g*_*e*_) and hole (*g*_*h*_) *g*-factors as a function of angle *θ* between the C-axis and the applied magnetic field. Right panels describe the electron (*m*_*ce*_) and hole (*m*_*ch*_) cyclotron masses.

The derivations can be found in the Methods section. We plot in Fig. 2 the *g*-factors and the effective cyclotron mass, 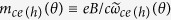 as a function of *θ*. At *θ* = 0, 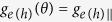 and *m*_*ce*(*h*)_(*θ*) = *m*_*e*(*h*)⊥_. At *θ* = *π*/2, *g*_*e*(*h*)_(*θ*) = *g*_*e*(*h*)⊥_ and 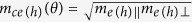.

### Rashba splitting

**Figure 3:**
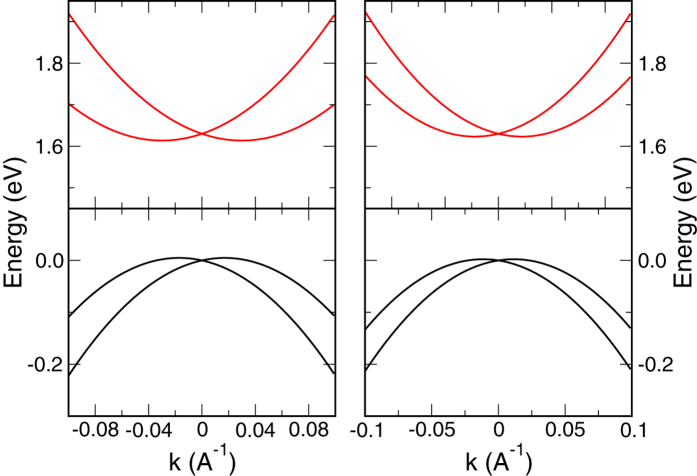
Rashba effect in the conduction and valence bands near the *R* point. Left (right) panel is for ***k*** along the [0, 1, 1] ([0, 1, 1]) direction. *ζ* = 0.5 eV.

The Rashba term, *E*_*c*(*v*)*r*_(*k*) = *α*_*c*(*v*)*r*_(*k*_*y*_*σ*_*x*_ − *k*_*x*_*σ*_*y*_) in Eqs (9) and (10), destroys the spin degeneracy, giving rise to energy-momentum dispersions, 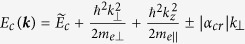 for the conduction band and 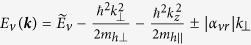 for the valence band, as plotted in Fig. 3.

The Rashba strengths *α*_*c*(*v*)*r*_ are directly related to the *C*_4*v*_ potential *ζ*-parameter that characterizes the inversion asymmetry of the structure,


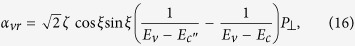



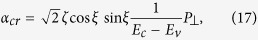


which indicate that the Rashba splittings in the valence and conduction bands are correlated. Currently the Rashba splittings obtained from different first-principles calculations vary significantly and direct measurements, such as spin-polarized photoemission and spin-flip Raman scattering, of CH_3_NH_3_PbI_3_ are not yet available. For *ζ* = 0.5 eV, we have *α*_*vr*_ = 0.565 and *α*_*cr*_ =1.088 eVÅ.

### Exciton wavefunctions

Hybrid perovskite CH_3_NH_3_PbI_3_ has a large dielectric constant *ε*, and the excitons are of the Wannier type, whose wave functions can be written as^35^


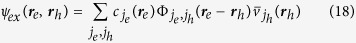


where *j*_*e*_, *j*_*h*_ = ±, 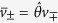 with 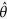 being the time-reversal operator. 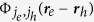 is the envelop function describing the relative motion of electron and hole and may have *S*, *P*, or *D* characteristics in low magnetic fields, which gradually transforms to that of Landau wave functions with increase of magnetic field.

The hole (electron) state 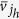 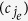 follows the 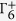 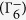 representation of *C*_4*v*_. With 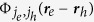 being the 1*S* state, the exciton wavefuctions can be characterized by the *C*_4*v*_ representations, 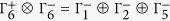,


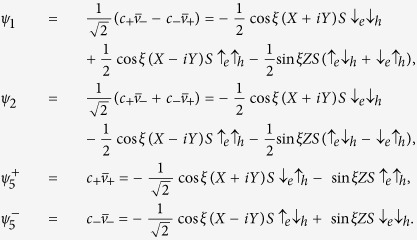


The total angular momentum ***J*** = ***j***_*e*_ + ***j***_*h*_ is *J* = 0 for *ψ*_1_, (*J*, *J*_*z*_) = (1, 0) for *ψ*_2_, and (*J*, *J*_*z*_ = 1, ±1) for 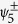. The absorption and emission of these states are proportional to the modular square of their electric-dipole elements,


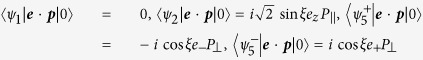


with 
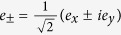
. Hence *ψ*_2_ can absorb and emit light polarized along the *z*-axis, and 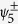 can absorb and emit light circularly polarized in the *x*-*y* plane. *ψ*_1_, however, is dark, for it contains only spin triplets and is therefore dipole-forbidden.

The above selection rules in the hybrid perovskites is in stark contrast to those in *π*-conjugated organic materials with weak SOCs^36^, where the electron (hole) has zero angular momentum (*l*_*z*_ = 0 for *π* orbitals) and *J* = 1 is dipole forbidden whereas *J* = 0 is dipole allowed. As we will see below, this difference gives rise to a far richer physics of MFE in the hybrid perovskites.

### Paramagnetic effects on excitons

The four 1*S* exciton states, *ψ*_1_, *ψ*_2_, and 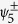, in general, are not degenerate in energy because of possible exchange interaction between spins 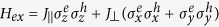, where ***σ***^*e*^/2 = ***j***_*e*_ and ***σ***^*h*^/2 = ***s***_*h*_. Consequently the energies of these excitons 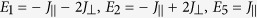.

An applied magnetic field can modify the exciton energy via the Zeeman energy,


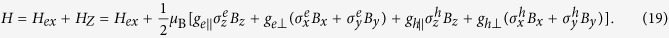


It should be noted that from the time reversal symmetry, the hole’s *g*-factor, including the sign, is identical to that of the valence electron. Since we are concerned with relatively weak magnetic field, we temporarily neglect the diamagnetic effect, which is proportional to *B*^2^ and shift states equally in energy.

In the Faraday configuration with ***B*** along the C-axis, ***B*** = (0, 0, *B*), 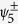 will split in energy, 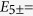

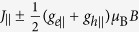
, and the splitting in absorption and luminescence peaks of left- and right-circularly polarized light, would be 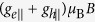
11,33,34.

**Figure 4:**
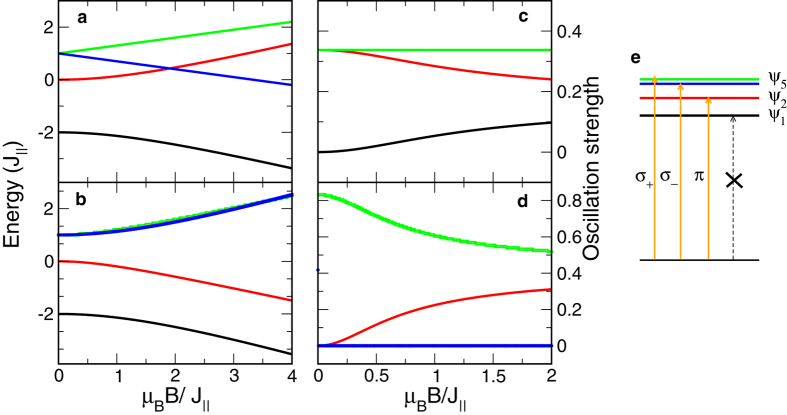
Exciton energies and oscillator strengths vs magnetic field. Panels (a,b) delineate exciton energies as a function of magnetic field in the Faraday and Voigt configurations, respectively. Panels (c,d) are the oscillator strengths of these exciton states along the *z*-axis and the *x*-axis, respectively. Panel (e) illustrates the electric-dipole selection rule of the exciton states. Black and red lines correspond to *ψ*_1_ and *ψ*_2_ excitons. Green and blue lines correspond to 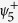 and 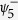 excitons in (**a**,**c**), and *ψ*_5*L*_ and *ψ*_5*T*_ in (**c**,**d**). Green and blue lines in (**c**) as well as black and blue lines in (**d**) are on top of each other. 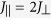.

The magnetic field ***B*** will also mix *ψ*_1_ and *ψ*_2_, with


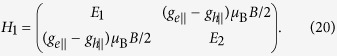


and the energies of eigenstates become 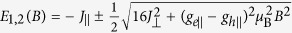 and their wave functions are 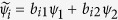 (*i* = 1, 2). Hence, with increase of the magnetic field, 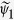 will gain oscillator strength, 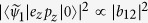, and flare up, while 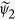 will lose oscillator strength, 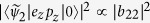, as illustrated in Fig. 4.

In the Vogit configuration with ***B*** perpendicular to the C-axis, ***B*** = (*B*, 0, 0), we can construct transverse and longitudinal states out of 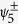 states, 
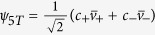
 and 
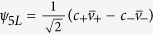
, which have polarization along the *y* and *x* axis, respectively. It is readily to verify that in this configuration, pair (*ψ*_1_, *ψ*_5*L*_) as well as pair (*ψ*_2_, *ψ*_5*T*_) are coupled via the magnetic field, with


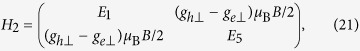



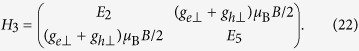


and their energies are 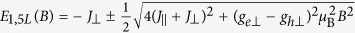 and 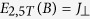

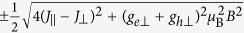
. Figure 4 also plots the exciton energies *E*_1_, *E*_2_, *E*_5*L*_, and *E*_5*T*_, as well as their corresponding oscillator strengths |〈*e*_*x *_*p*_*x*_〉|^2^ as a function of the magnetic field.

### Magnetic-field effect on photoluminescence

The PL intensity in CH_3_NH_3_PbI_3_ is found to be susceptible to a magnetic field at low temperatures^11^. In the Faraday configuration, the magnetic field couples *ψ*_1_ and *ψ*_2_ excitons. Since only the recombination of *ψ*_2_ can give rise to luminescence, the magnetic-field-induced change in populations of *ψ*_2_ and *ψ*_1_ would lead to an MFE. We employ the Bloch equation of the density matrix to systematically describe the population dynamics,


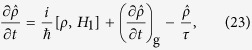


where 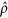 is a 2 × 2 density matrix spanned by *ψ*_1_ and *ψ*_2_, 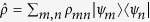 with *m*, *n* = 1, 2. (∂*ρ*/∂*t*)_g_ represents the generation of the exciton states, which is finite only for diagonal terms, (∂*ρ*_*mn*_/∂*t*)_g_ = *F*_*m*_*δ*_*mn*_, because the PL in the MFET measurements^11^,^12^ is not resonantly excited. *τ* is the relaxation time of these exciton states, which includes both recombination 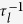 and spin relaxation 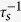, 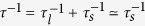, for 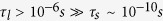
5,6,11. In the steady state, 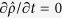, the densities at *ψ*_1_ and *ψ*_2_ can be written as 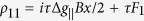 and 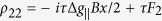, where 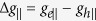 and 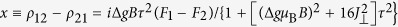. The intensity change in PL is due to the change in *ρ*_22_,


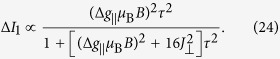


When the exchange is significant with 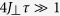, 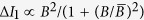 with 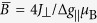. In this regime, the MFE is suppressed because the magnetic field cannot overcome the exchange to effectively alter the populations on the exciton states for *H* < 1T. When 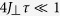, the denominator in Eq. (24) becomes 
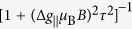
, which represents the so called Δ*g* mechanism of MFE. The Δ*g* mechanism has been found responsible for many MFE phenomena involving radical pairs in organic systems^24^. If the 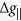 is known, the Lorentz line shape in Eq. (24) can be used to measure the exciton relaxation time *τ*, or equivalently, its spin relaxation time.

In the Vogit configuration, the MFE in PL is also expected because the magnetic field, as shown in Eqs (21) and (22), mixes the dark *ψ*_1_ with the dipole-allowed *ψ*_5*L*_, and *ψ*_2_ with *ψ*_5*T*_, which have different oscillator strengths and polarizations, 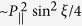 and along the *z*-axis and 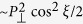 and along the *y*-axis for *ψ*_5*T*_, respectively. Using the Bloch equation, we express the PL change in the *ψ*_1_ and *ψ*_5*L*_ manifold as


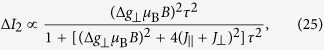


where Δ*g*_⊥_ = *g*_*e*⊥_ − *g*_*h*⊥_, and the PL change in the manifold of *ψ*_2_ and *ψ*_5*T*_ as


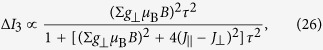


where Σ*g*_⊥_ = *g*_*e*⊥_ + *g*_*h*⊥_. The MFE in the *ψ*_1_ and *ψ*_5*L*_ manifold, Δ*I*_2_, depends on the difference in the *g*-factors along the *x*-axis, the direction of the magnetic field, in a very similar fashion as Δ*I*_1_.

The MFE in the *ψ*_2_ and *ψ*_5*T*_ manifold, Δ*I*_3_, however, depends on the summation of the electron and hole *g*-factors along the *x*-axis. Thus in addition to the Δ*g* mechanism, a Σ*g* mechanism is taking effect in the hybrid perovskites. In the former, the magnetic field modulates the populations between states *J* = 0 and (*J*, *J*_*z*(*x*)_) = (1, 0), which can be visualized as the electron and hole spins precess along the magnetic field in the *opposite* directions. In the latter, the magnetic field modulates populations between (*J*, *J*_*z*_) = (1, 0) and (1, ±1), which can be visualized as the electron and hole spins precess along a transverse magnetic field in the *same* direction. The Σ*g* mechanism is particularly important if the exchange is approximately isotropic, 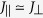, where the *J* = 1 triplet states are degenerate in energy, and according Eq. (26), Δ*I*_3_ is then completely determined by the exciton relaxation time *τ*, whereas Δ*I*_1_ and Δ*I*_2_ in Eqs (24) and (25) are suppressed by the exchange splitting 4*J*_⊥_ between the singlet and triplet. For polycrystalline materials, the intensity change should be the combination of the three processes, Δ*I*_*i*_ (*i* = 1, 2, 3), suggesting that multiple Lorentzen functions may be required to describe experimental data, as shown experimentally^11^.

### Photoexcitation intensity dependence of MFE

**Figure 5:**
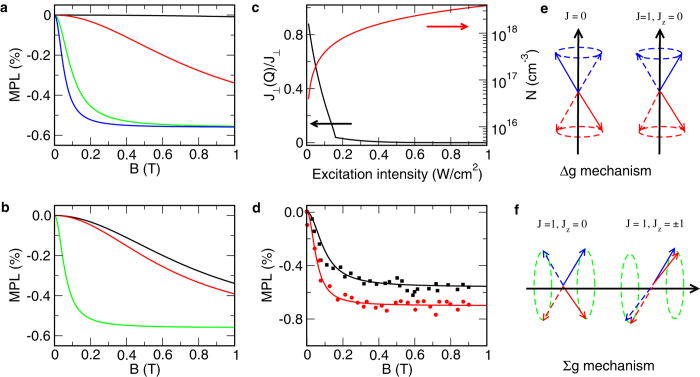
Magnetic field effect in exciton photoluminescence. Panel (a) describes Δ*I*_1_ for exchange splitting between *ψ*_2_ and *ψ*_1_ being 4*J*_⊥_ = 1 (black line), 0.1 (red line), 0.01 (green line), and 0.001(blue line) meV. Panel (b) describes Δ*I*_1_ (black line), Δ*I*_2_ (red line), and Δ*I*_3_ (green line) for 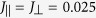 meV. Panel (c) describes the carrier density and the reduction of the exchange as a function of the intensity of excitation light. Panel (d) plots Δ*I*_1_ as a function of magnetic field with 4*J*_⊥_ = 1 meV for intensities of 0.34 (black line) and 0.85 W/cm^2^ (red line). Black and red dots are the corresponding experimental values in ref. 12. Panels (e,f) illustrate the Δ*g* and Σ*g* mechanisms. The exciton relaxation time is fixed at 10^−10^ s.

It is observed that the MFE in PL in CH_3_NH_3_PbI_3_ is also dependent on the photoexcitation intensity^12^. Only when the intensity reaches a certain threshold, does the MFE become significant. The line shape of MFE shrinks with the increase of the photoexcitation intensity and is eventually stabilized. To explain this unusual intensity dependence, we notice that the MFE, as shown in Fig. 5, is pronounced only when the Zeeman energy dominates over the exchange splitting. As a specific example, we consider the Faraday configuration, where the energy splitting between *ψ*_1_ and *ψ*_2_ is 4*J*_⊥_. This exchange is of short-range and related to the exciton envelop function Φ(***r***) at *r* = 0, i.e., when the electron and hole are at the same location. For the 1*S* state,


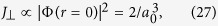


where *a*_0_ is the effective Bohr radius of the exciton, *a*_0_ = *ħ*^2^*ε*/*e*^2^*μ* with 
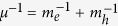
 being the effective mass of exciton.

A high-intensity photoexcitation creates many free electron-hole pairs, whose density can be estimated as *N* = *αIτ*_*l*_/*ħω*, where *α* is the absorption coefficient, *α* ~ 10^5^ cm^−1 ^5 for the CH_3_NH_3_PbI_3_, *τ*_*l*_ is the carrier recombination lifetime, 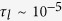 s, and *ħω* is the photon energy. These free electron-hole pairs will screen the Coulomb interaction, which can be modeled by the Debye-Hückel theory of ion gases^37^. The Coulomb potential −*e*^2^/*εr*, in the presence of charged particles, is replaced by the potential *U* that satisfies the Poisson equation, (∇^2^ − *Q*^2^)*U* = 0 with *Q*^2^ = 8*πe*^2^/*k*_*B*_*TN*. The solution *U*(*r*) is of the Yukawa type *U*(*r*) = −*e*^2^*e*^−*Qr*^/(*εr*), and the ground-state wave function in such a potential can be written as 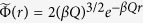, where the trial parameter *β* can be obtained by minimizing the ground state energy of Hamiltonian −∇^2^/2*μ* + *U*(*r*), *E* = (1)/(2)*β*^2^*Q*^2^*ħ*^2^/*μ* − 4*β*^3^*e*^2^/[*εQ*(4*β*^2^ + 4*β* + 1)]. The wave function 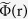, as compared to Φ(r), is more spreaded in space, and the exchange will be reduced by a factor


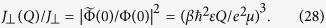


Figure 5 illustrates the screening effect. We see that as the intensity of photoexcitation increases, the exchange is greatly reduced. The MFE, meanwhile, becomes significant. After the carrier density reaches 10^18^ cm^−3^, the exchange is so small that 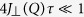, and the line shape in Δ*I*_1_ is independent of exchange, and therefore the photoexcitation intensity.

### Diamagnetic effect on excitons

So far we have considered only the 1*S* exciton states and neglected the diamagnetic effect on excitons. The diamagnetic effect originates from the orbital motions of electron and hole, and has been used to directly measure the exciton’s binding energy and effective mass^23^. For free electrons and holes, an applied magnetic field can localized their orbital wave function normal to the magnetic field, forming the Landau levels with the magnetic length of 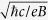. Since the anisotropy in effective mass for both electron and hole are relatively small, as shown in Fig. 2, we use isotropic effective masses, 
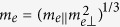
, m_h_ =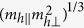, and 
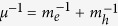
, to study the diamagnetic effect.

The diamagnetic response of excitons is far more complex than that of free electron-hole pairs, but offers more valuable information on excitons as well as constituent electrons and holes. Under a small magnetic field, the Coulomb interaction in an exciton is predominant and the diamagnetic effect can be studied by applying the perturbation theory to the hydrogen-like exciton wavefunctions. Such a perturbation must fail when the magnetic length 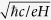 becomes much smaller than the orbital radius of exciton, *a*_0_ = *ħ*^2^*ε*/(*e*^2^*μ*). In this high-magnetic field regime, it is more appropriate to use the Landau levels as the starting point. A ratio, *γ* = *ħω*_*c*_/2*R*_y_, between the exciton cyclotron energy *ħω*_*c*_ = *eB*/(*μc*) and the exciton binding energy, *R*_y_ = *μe*^4^/(2*ε*^2^*ħ*^2^), can be used to distinguish the weak (*γ* < 1) and strong (*γ* > 1) magnetic-field regimes.

The Hamiltonian for the envelop function Φ(***r***_*e*_ − ***r***_*h*_) at *K* = 0 (*K* being the center-of-mass momentum of exciton) reads


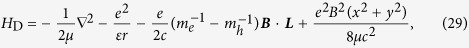


where ***L*** = −*i**r*** × ∇ is the orbital angular momentum. While this Hamiltonian of exciton is similar as that of hydrogen atom in a magnetic field, the key difference is that the third term contains 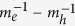, the difference between electron and hole effective masses, which will reduce to *μ*^−1^ in the hydrogen case.

To reliably solve the Hamiltonian for an arbitrary magnetic field, we employ two different basis sets^38^. In low magnetic fields, by using *a*_0_ (*R*_y_) as the length (energy) scale, 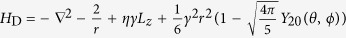, where *η* = *m*/*m*_*e*_ − *m*/*m*_*h*_ and *Y*_*lm*_ is spherical harmonic function. We choose the basis set as


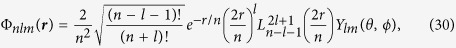


which are the eigenstates of 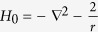 with 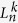 is the generalized Laguerre polynomials.

In high magnetic fields, by using 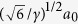 and 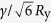 as the length and energy scales 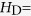

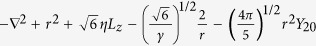
, and the eigenfunctions of a spherical harmonic oscillator *H*_0_ = −∇^2^ + *r*^2^


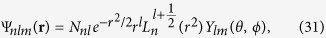


with 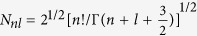 are chosen to be another basis set. These basis sets, with correct characteristics of wave functions in the low- and high-field regimes, facilitate an analytical evaluation of the Hamiltonian matrix elements (see the Methods section). Moreover the basis sets are eigenstates of parity and *L*_*z*_ = *m*, which are good quantum numbers of the Hamiltonian. We use large basis sets in both regimes, *n*, *l* ≤ 20 for Φ_*nlm*_ and *n*, *l* ≤ 29 for Ψ_*nlm*_, and diagonalize the Hamiltonian to obtain the eigenstates. The large basis sets allow us to approach the intermediate *γ* ~ 1 from both *γ* < 1 and *γ* > 1 regimes so that the solutions from both ends are smoothly connected. In the limit of *γ* → ∞, the eigenstates become the the Landau levels of free electron and hole. 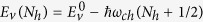 and 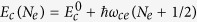. The optical selection rule for the transition from valence- to conduction-band Landau levels is *N*_*h*_ = *N*_*e*_ = *N*, and the absorption peaks are located at 
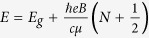
, where *E*_*g*_ is the band gap.

**Figure 6:**
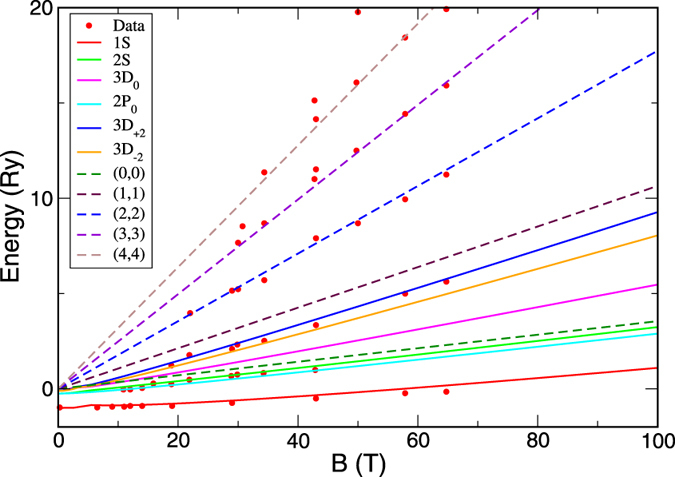
Cyclotron resonance of excitons in CH_3_NH_3_PbI_3_. Solid lines are energies of different exciton states. Dashed lines are the energy difference between the Landau levels of free electron and holes with (*N*, *N*) being the Landau-level index. Dots are the experimental data from ref. 23. The absorption peak between 2*S* and (1, 1) coincides with the energies of 3*D*_±2_ states.

In Fig. 6, we compare the theoretical results with recent cyclotron resonance experiment^23^, using the effective masses of *m*_*h*_ = 0.223 *m* and *m*_*e*_ = 0.188 *m*, and the binding energy of 16 meV. The agreement between theory and experiment is excellent. In addition, the pronounced absorption peaks above the 2*S* state, induced by the magnetic field, are very close to the state 3*D*_±2_, whereas the 2*P*_0_ state, as assigned in ref. 23 for the absorption peak, is almost degenerate in energy with 2*S* state. Indeed, transitions to exciton states 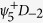 and 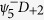, according to the selection rule, are electric-dipole allowed and the only reason that these the state is dark at zero field is Φ(*r* = 0) = 0, which become finite in large magnetic fields. Thus we believe that the observed excitonic absorption peak is due to the 3*D*_±2_ states. 3*D*_±2_ states are important in that from their energy splitting, 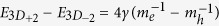, we can obtain the difference in the effective masses of electron and hole, which, together with *μ* measured from the Landau levels of free electron-hole absorption, can completely determine both *m*_*e*_ and *m*_*h*_. It is noted that the inter-band cyclotron resonance of free electron-hole pairs can measure only *μ*, not individual *m*_*e*_ and *m*_*h*_. Thus cyclotron resonance of excitons reveals more information.

## Discussion

The hybrid inorganic-organic perovskites have shown great promise in photovoltaic and many other important applications because of their outstanding transport, optical, and magneto-optical properties. To understand these properties, in this paper, we have constructed a reliable and accessible effective-mass model of the hybrid perovskites, which connects effective masses, Rashba splittings, and anisotropic *g*-factors of conduction and valence bands. Using this effective-mass model, we have elucidated the observed MFE in exciton PL and its dependence on photoexcitations and identified a new Σ*g* mechanism of MFE. We have also calculated the cyclotron resonance of excitons for arbitrarily strong magnetic fields and pointed out that excitonic states such as 3*D*_±2_ provide information on the difference in effective masses of electron and hole.

This effective-mass model is a foundation on which systematic models of electron-phonon coupling, carrier mobility, and other transport properties can be developed. Because of the concise expressions of SOC, Rashba effect, and *g*-factor, this model also facilitates studies of spin relaxation^39^, spin Hall effect^40^, and other magneto optical and spintronic phenomena.

## Methods

### Expressions of *H*_A_, *H*_B_, and *H*_C_

The three 4 × 4 matrices in Eq. (7) are displayed as


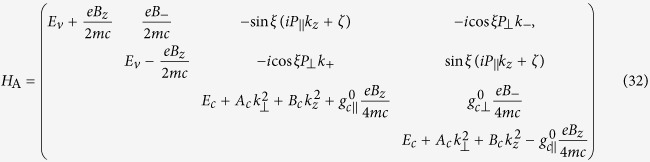


where


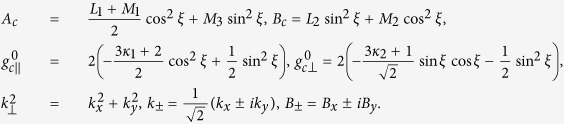



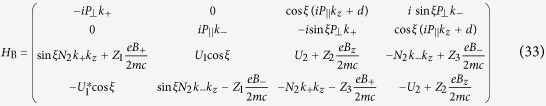


where


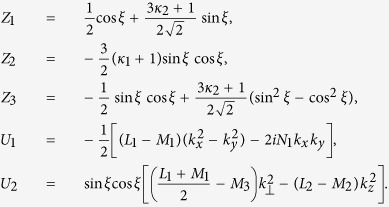



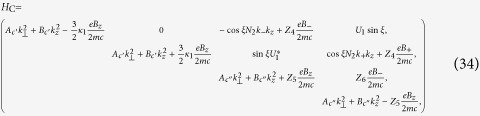


where


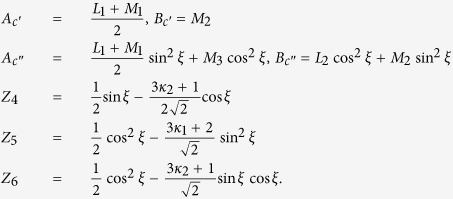


### Derivations of Eq. (15)

When the applied magnetic field ***B*** tilts away from the crystal C-axis with an angle *θ*, we can define the new *z*-axis (denoted *z*′) along ***B*** and assume that the tilting is in the *x*-*z* plane with the new *x*-axis denoted as *x*′. The transformations of coordinates between the two references are


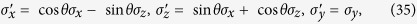



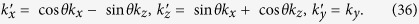


The Zeeman energy in Eqs (9) and (10) then becomes


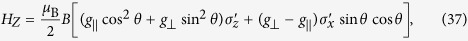


and the effective *g*-factor *g*(*θ*) in Eq. (15) can be obtained by diagnonalizing this Hamiltonian.

In the new reference system, 
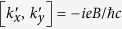
, and the kinetic energy in Eqs (9) and (10) reads


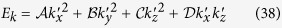


with 
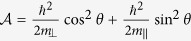
, 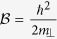 
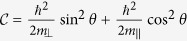
, 
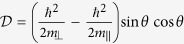
. We can define the ladder operators *b* and *b*^+^ of the Landau levels as


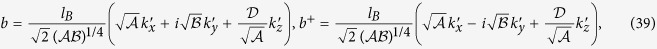


with 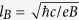, which satisfy [*b*, *b*^+^] = 1. The kinetic energy is then expressed as


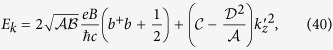


with 
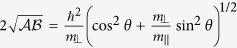
, which gives *m*_*c*_(*θ*) in Eq. (15).

### Matrix elements of *H*_D_ in basis sets of Φ_*nlm*_ and Ψ_*nlm*_

The matrix elements of *H*_D_ among Φ_*nlm*_ and among Ψ_*nlm*_ can be evaluated analytically, which greatly simplifies the eigenstate calculations.

The matrix element of angle-dependent term in the Hamiltonian can be calculated via the integral


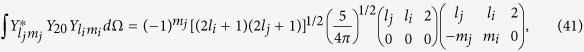


where the Wigner 3-*j* symbols are used.

The only type of matrix elements to be evaluated is *r*^*s*^ between the basis functions. Between basis functions 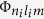 and 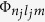,


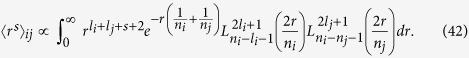


The integral can be worked out analytically,


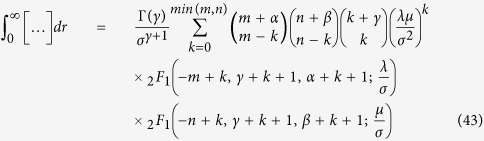


where *γ* = *l*_*i*_ + *l*_*j*_ + *s* + 2, 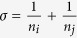, *α* = 2*l*_*i*_ + 1, *β* = 2*l*_*j*_ + 1, *m* = *n*_*i*_ − *l*_*i*_ − 1, *n* = *n*_*j*_ − *l*_*j*_ − 1, *λ* = 2/*n*_*j*_, *μ* = 2/*n*_*j*_, and _2_*F*_1_(*α*, *β*, *γ*; *z* is the Gauss’ hypergeometric function.

The matrix element of *r*^*s*^ between basis functions 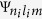 and 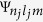, neglecting the normalization factor, is


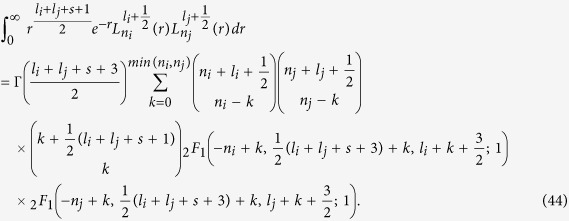


## Additional Information

**How to cite this article**: Yu, Z. G. Effective-mass model and magneto-optical properties in hybrid perovskites. *Sci. Rep.* **6**, 28576; doi: 10.1038/srep28576 (2016).
